# Calcium in the Backstage of Malaria Parasite Biology

**DOI:** 10.3389/fcimb.2021.708834

**Published:** 2021-07-28

**Authors:** Lucas Silva de Oliveira, Marcos Rodrigo Alborghetti, Renata Garcia Carneiro, Izabela Marques Dourado Bastos, Rogerio Amino, Philippe Grellier, Sébastien Charneau

**Affiliations:** ^1^Laboratory of Biochemistry and Protein Chemistry, Department of Cell Biology, Institute of Biology, University of Brasilia, Brasilia, Brazil; ^2^UMR 7245 MCAM, Molécules de Communication et Adaptation des Micro-organismes, Muséum National d’Histoire Naturelle, CNRS, Équipe Parasites et Protistes Libres, Paris, France; ^3^Brazilian Biosciences National Laboratory, Brazilian Center for Research in Energy and Materials, Campinas, Brazil; ^4^Laboratory of Host-Pathogen Interaction, Department of Cell Biology, Institute of Biology, University of Brasilia, Brasilia, Brazil; ^5^Unité Infection et Immunité Paludéennes, Institut Pasteur, Paris, France

**Keywords:** Ca^2+^ signaling, *Plasmodium*, intracellular messenger, homeostasis, invasion, egress

## Abstract

The calcium ion (Ca^2+^) is a ubiquitous second messenger involved in key biological processes in prokaryotes and eukaryotes. In *Plasmodium* species, Ca^2+^ signaling plays a central role in the parasite life cycle. It has been associated with parasite development, fertilization, locomotion, and host cell infection. Despite the lack of a canonical inositol-1,4,5-triphosphate receptor gene in the *Plasmodium* genome, pharmacological evidence indicates that inositol-1,4,5-triphosphate triggers Ca^2+^ mobilization from the endoplasmic reticulum. Other structures such as acidocalcisomes, food vacuole and mitochondria are proposed to act as supplementary intracellular Ca^2+^ reservoirs. Several Ca^2+^-binding proteins (CaBPs) trigger downstream signaling. Other proteins with no EF-hand motifs, but apparently involved with CaBPs, are depicted as playing an important role in the erythrocyte invasion and egress. It is also proposed that a cross-talk among kinases, which are not members of the family of Ca^2+^-dependent protein kinases, such as protein kinases G, A and B, play additional roles mediated indirectly by Ca^2+^ regulation. This statement may be extended for proteins directly related to invasion or egress, such as SUB1, ERC, IMC1I, IMC1g, GAP45 and EBA175. In this review, we update our understanding of aspects of Ca^2+^-mediated signaling correlated to the developmental stages of the malaria parasite life cycle.

## Introduction

A plethora of cell types employ the calcium ion (Ca^2+^), mobilized from extracellular and/or intracellular environments, to coordinate different Ca^2+^-dependent processes. The control of intracellular Ca^2+^ signals is dynamic. Overall, fluctuations in Ca^2+^ concentrations are modulated by an influx and/or efflux promoted by membrane channels, such as store-operated calcium channels (SOCEs), plasma membrane Ca^2+^-ATPase (PMCA) and sarco(endo)plasmic reticulum Ca^2+^-ATPase (SERCA) pumps. Generally, this orchestration in the Ca^2+^ concentration follows a signaling pathway that obeys the order: stimuli, G-protein coupled receptor, phospholipase C (PLC) activation, mobilization of phosphatidylinositol 4,5-biphosphate (PI(4,5)P2), production of inositol 1,4,5-phosphate (IP3), IP3 recognition by IP3-sensitive receptor channels (IP3Rs) in the endoplasmic reticulum (ER) and downstream Ca^2+^ cascade (Berridge et al., 2003; Clapham, 2007).

In the group of apicomplexan parasites, the protozoan parasites *Toxoplasma gondii* and *Plasmodium* spp. are the most well-established study models. In *T. gondii*, Ca^2+^ signaling is involved in specific parasite processes: motility, conoid extrusion, attachment, invasion and egress from the host cell (Borges-Pereira et al., 2015; Hortua-Triana et al., 2018). Similarly, Ca^2+^ homeostasis and signaling have been extensively studied in *Plasmodium* species. Malaria is still the most life-threatening vector-borne disease globally, with an estimated 409,000 deaths and 229 million cases reported in 2019 (Global Malaria Programme: WHO Global, 2020). The increase and dissemination of antimalarial resistance (Cowman et al., 2016; Phillips et al., 2017; Global Malaria Programme: WHO Global, 2020), together with the augmentation of malaria cases since 2015, point to an urgent need for the discovery of new antimalarial drugs. The *Plasmodium* life cycle is strongly regulated by fluctuations in Ca^2+^ cellular levels, with deficiency causing impairment in parasite growth and invasion rate (Wasserman et al., 1982). This ion also acts as a messenger regulating critical *Plasmodium* biological processes. As such, proteins involved in Ca^2+^ homeostasis and signaling are strong candidates as new antimalarial targets (Gazarini et al., 2007; Vidadala et al., 2014; Mossaad et al., 2015; Bansal et al., 2016; Fang et al., 2017; Iyer et al., 2018). In this review, we present an overview of the mechanisms related to the Ca^2+^ homeostasis in *Plasmodium* species and an update of the main downstream Ca^2+^ signaling pathways and effectors involved in the parasite motility, invasion, development, and egress.

## Ca^2+^ Homeostasis in Malaria Parasites

Ca^2+^ signaling is widely conserved in Eukaryotes, with reliance on this ion as a secondary messenger to switch on or off diverse biological process. Given their evolutionary distance from other Eukaryotes, malaria parasites represent a challenging task for the study of Ca^2+^-mediated mechanisms, with Ca^2+^ uptake by this microorganism presenting several peculiar features. Since *Plasmodium* asexual developmental stages are predominantly intracellular in red blood cells (RBCs), Ca^2+^ has to cross several barriers to reach the parasite, which include the red blood cell membrane (RBCM) and parasitophorous vacuole membrane (PVM) (Kirk, 2001; Kirk, 2004; Kirk and Lehane, 2014).

Ca^2+^ fluctuations in *Plasmodium* species are very complex and demand the support of intracellular Ca^2+^ storage. For example, gametocytes or schizont fractions from *Plasmodium chabaudi* infected RBCs (iRBCs) present 10-20 times more Ca^2+^ than uninfected RBCs. Moreover, it has been observed that this ion concentrates in parasite storage compartments (Tanabe et al., 1982). This pattern of Ca^2+^ concentration was also observed in *Plasmodium falciparum* (Adovelande et al., 1993). To overcome these barriers and promote the observed intracellular Ca^2+^ increase, malaria parasites facilitate RBCM permeability, causing increased Ca^2+^ influx and decreased Ca^2+^ efflux (Tanabe et al., 1982; Desai et al., 1996). A nonselective cation conductance at RBCM, induced by *P. falciparum* growth, has been proposed as a mechanism involved in Ca^2+^ permeability (Brand et al., 2003; Duranton et al., 2003). Furthermore, Na^+^ associated to Ca^2+^ influx is also involved in intracellular parasite growth by this mechanism, potentially involving an ethylisopropyl-amiloride (EIPA)-sensitive channel (Brand et al., 2003).

In addition to the RBCM, PVM is another barrier to Ca^2+^ reaching the *Plasmodium* parasite. Using a cell-attached path clamp method, a 140-pS channel that is permeable to Ca^2+^, other ions and nutrients was identified and proposed to mediate this transport through the PVM (Desai et al., 1993). Despite such advances, mechanisms involving Ca^2+^ transport into malaria parasites remain poorly understood, with considerable attention now given to this area with regard to potential therapeutic intervention. Blocking the *Plasmodium* translocon for exported proteins machinery (PTEX)‐mediated protein export across the PV and out into the RBC cytosol by conditional knockdown approach, significantly reduced Ca^2+^ permeability in iRBCs (Kushwaha et al., 2018), revealing that exported parasite proteins are potentially involved in Ca^2+^ uptake and transport.

Determination of the concentration of intracellular Ca^2+^ in apicomplexan parasites is still controversial, primarily because of the technical limitations due to inhibitors, ionophores and fluorometric measurement assay sensitivities. It is widely accepted that the intracellular concentration of Ca^2+^ is around 0.09-0.1 µM in physiological conditions, similar to those found in other Eukaryotes (Alleva and Kirk, 2001; Moreno et al., 2011; Lourido and Moreno, 2015). Nonetheless, an increase of up to a hundred-fold in Ca^2+^ concentrations was noted in the late stage of the intraerythrocytic cycle forms, ranging from 1-10 µM (Glushakova et al., 2013). Also, a high Ca^2+^ concentration (40 µM) was reported in the parasitophorous vacuole (PV) required for proper parasite development (Gazarini et al., 2003). More recently, by using the Ca^2+^ sensor yellow cameleon (YC)-Nano, dynamic measurement of intracellular Ca^2+^ in different life stages of *P. falciparum* shows significant fluctuations throughout the parasite development: ring (~370 nM), trophozoite (~30 nM), schizont (~310 nM), merozoite (~950 nM), and gametocyte (stage III, ~130 nM, stage IV-V, ~520 nM) stages (Pandey et al., 2016).

Actors modulating such Ca^2+^ fluctuations have now begun to be identified, although it is still a subject under debate. For example, cytoplasmic Ca^2+^ increase may be related to potassium (K^+^) availability, especially when parasites are faced with an abrupt change from high to low K^+^ concentration. Exposition of *P. falciparum* merozoites to an ionic environment with a low K^+^ concentration (which is the environment usually found by parasites after egress from RBCs) increases the levels of cytosolic Ca^2+^ (Singh et al., 2010). This leads to the production of cyclic-adenosine monophosphate (cAMP) by PfACβ (adenylyl-cyclase β) upon $HCO_{3}^{-}$ (bicarbonate ions) stimulation, followed by activation of protein kinase A (PKA) and microneme secretion (Dawn et al., 2014; Kumar et al., 2017). However, exactly how K^+^ acts on signaling for merozoite maturation and invasion is controversial, in contrast to intracellular cationic remodeling in iRBC (Pillai et al., 2013).

In addition, it was also demonstrated that a putative and conserved protein member from the Epac (exchange protein directly activated by cAMP, PF3D7_1417400) pathway in *P. falciparum* is potentially involved in the rise of cytosolic Ca^2+^ levels, facilitating *P. falciparum* merozoite invasion by triggering microneme secretion (Dawn et al., 2014). Nonetheless, this pathway is apparently not required for parasite growth and egress (Patel et al., 2019). Moreover, key elements in this Ca^2+^ mobilization were shown to involve the serpentine GPCR-like receptor *Pf*SR25, a monovalent cation sensor coupled to PLC in triggering the cytoplasmic Ca^2+^ increase. Data also support the involvement of PfSR25 in parasite stress survival (Moraes et al., 2017).

**Figure 1:**
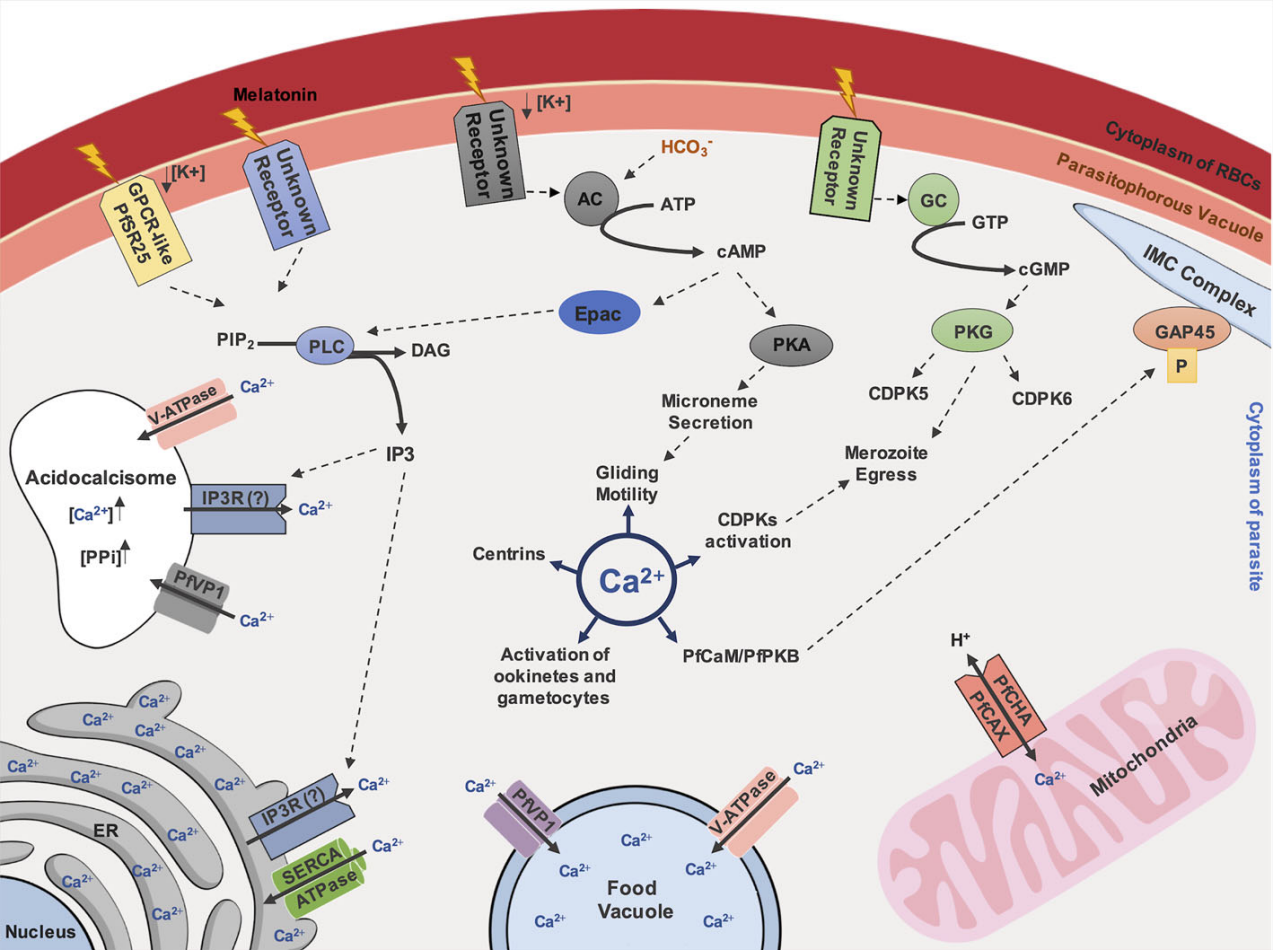
Ca^2+^-dependent signaling pathway in *Plasmodium* species. Ca^2+^ inside the cytoplasm of parasite controls important processes for parasite survival, such as gliding motility, mediated by activation of PfCaM/PfPKB complex and following phosphorylation of the IMC member protein, GAP45. Additionally, centrins, CDPKs activation, activation of ookinetes and gametocytes are described as Ca^2+^-regulated. A GPCR-like protein, named as PfSR25, has been described as potential regulator in Ca^2+^ homeostasis in malaria parasites, depending on availability of potassium (K^+^) and mediated by IP3 signaling. Melatonin was also described as a trigger for IP3 dependent pathways. Endoplasmic Reticulum (ER) is reported as the major storage of Ca^2+^ and the uptake of this ion possibly depends on SERCA-type Ca^2+^-ATPases. Ca^2+^ discharge depends on receptors activated by IP3, nonetheless, an IP3R remains to be discovered in *Plasmodium* species. The presence of V-ATPase and VP1 on the food vacuole and acidocalcisome membranes are related to the Ca^2+^ uptake upon an acidic environment maintenance. Acidocalcisome could also have an IP3R that allows exit of Ca^2+^. Calcium can also enter in mitochondria through Ca^2+^/H^+^ antiporter called PfCHA/PfCAX. Activation of PKA and PKG, by cAMP and cGMP, respectively, generated by adenylyl-cyclase (AC) and guanylyl-cyclase (GC), respectively, could also participate in Ca^2+^ homeostasis, however the membrane receptors that stimulate theses pathways remains to be elucidated. Still, upon HCO_3_¯ activation, AC can also stimulate Epac activation by cAMP, triggering IP3 signaling through PLC activation. Additionally, a cross-talk among kinases are also proposed to be associated to the merozoite egress mediated by proteolytic cascade events.

Host molecules can also modulate parasite Ca^2+^ levels. For example, melatonin, which appears as a critical signal controlling synchronous maturation of *Plasmodium in vivo*, triggers an increase in Ca^2+^ cytoplasmic concentration through Ca^2+^ release from intracellular stores by an IP3-dependent pathway activation (Gazarini et al., 2003; Beraldo et al., 2005; Beraldo et al., 2007; Alves et al., 2011; Pecenin et al., 2018). Under melatonin stimulation, Ca^2+^ mobilization is affected by the melatonin receptor antagonist luzindole, the PLC inhibitor U73122 (Hotta et al., 2000) and IP3 receptor blockers (2-APB, 2-aminoethyl diphenylborinate derivatives) (Beraldo et al., 2007; Pecenin et al., 2018). Together, these data support a complex Ca^2+^-signalling network in high demand for intraerythrocytic parasite development (**Figure 1**).

## Ca^2+^ Storage Organelles

### Endoplasmic Reticulum (ER)

The ER is the central organelle for Ca^2+^ storage, with a specific pathway to control calcium influx and efflux in the cell of apicomplexan parasites (Moreno et al., 2011; Lourido and Moreno, 2015). Both *P. falciparum* and *T. gondii* have Ca^2+^ pumps in the ER membrane, known as SERCA-type Ca^2+^-ATPases, that provide Ca^2+^ transport activity (Eckstein-Ludwig et al., 2003; Nagamune et al., 2007; Nagamune et al., 2008). *Pf*ATP6 is the only SERCA-type Ca^2+^-ATPase found in the *P. falciparum* genome (Gardner et al., 2002). Due to structural similarities to a SERCA inhibitor, known as thapsigargin (Thg), the antimalarial drug, artemisinin, was thought to act against *Pf*ATP6, occasionally inhibiting Ca^2+^ mobilization into ER. Initial evidence for this hypothesis were observed in *Xenopus* oocystes expressing different *P. falciparum* transporters, including *Pf*ATP6 (Eckstein-Ludwig et al., 2003). However, molecular docking and experimental validation assays showed that the interaction between *P. falciparum* SERCA (*Pf*SERCA) and dihydroartemisinin (dART) was ~2.3-fold weaker than those observed between human SERCA and dART, indicating that dART do not inhibit *Pf*SERCA pump activity, refuting the initial conclusion (Pandey et al., 2016).

Generally, Ca^2+^ mobilization from ER storage requires IP3 activation. The production of this molecular signal is provided by PLC (Singh and Chitnis, 2012; Brochet and Billker, 2016). Although IP3-mediated Ca^2+^ release from intracellular stores have been widely reported (Lovett et al., 2002; Alves et al., 2011; Glushakova et al., 2013; Pecenin et al., 2018; Borges-Pereira et al., 2020), no genetic information is known about the presence of IP3R in apicomplexan (Lourido and Moreno, 2015; Garcia et al., 2017). It has been widely accepted that a different IP3-dependent mechanism may exist in apicomplexan to mobilize Ca^2+^ from intracellular stores (Moreno et al., 2011; Lourido and Moreno, 2015). This statement is based on many reports, which have shown that upstream inhibition of the IP3 pathway by using PLC inhibitor (Hotta et al., 2000; Beraldo et al., 2005; Beraldo et al., 2007), and downstream inhibition by using IP3 receptor blocker (Beraldo et al., 2007; Pecenin et al., 2018) and SERCA inhibitor (Alves et al., 2011; Glushakova et al., 2013; Pecenin et al., 2018; Borges-Pereira et al., 2020), all lead to the blockage of Ca^2+^ mobilization in- or outward from the cytosolic environment or IP3-sensitive stores. Since there is no clear evidence that an IP3R exists at the ER in *Plasmodium* species, how Ca^2+^ mobilization occurs into this compartment and how the ER may contribute to Ca^2+^ homeostasis through an IP3-sensible mechanism are still unresolved.

### Acidic Organelles

Other Ca^2+^-storage organelles are described in Apicomplexans, such as acidocalcisomes and food vacuole (FV), which stock Ca^2+^ in an acidic environment (Lourido and Moreno, 2015). The acidocalcisome is a lysosomal-like compartment, rich in pyrophosphate (PPi), polyphosphate (PolyP) complexed with Ca^2+^ and other cations (Moreno and Docampo, 2009; Docampo and Huang, 2016). This organelle was observed in *P. falciparum* by Ruiz et al. (2004) after being described in other parasites, such as *Trypanosoma brucei* (Vercesi et al., 1994), *Trypanosoma cruzi* (Docampo et al., 1995) and *T. gondii* (Moreno and Zhong, 1996).

Two enzymes found in the *P. falciparum* genome, described as vacuolar-H^+^-pyrophosphatase (VP1) and vacuolar-H^+^-ATPase (V-ATPase), can use PPi and ATP, respectively, to pump protons toward the lumen of acidocalcisomes, providing acidification of the structures, supporting Ca^2+^-storage maintenance in these organelles (Docampo et al., 2005). VP1 and V-ATPase are also localized in the FV in *Plasmodium* species, suggesting this acidic compartment may also have a role in regulating Ca^2+^-storage (Saliba et al., 2003). The potential role of these acidic organelles in Ca^2+^ storage is supported by the V-ATPase and VP1 blockage in malaria parasites by their respective inhibitors, bafilomycin A_1_ and amino-methylene-diphosphonate (AMDP), causing an increase in cytosolic Ca^2+^ levels (Luo et al., 1999; Biagini et al., 2003).

While the FV in malaria parasites can store around 300-400 nM of Ca^2+^, this compartment is not considered a major intracellular Ca^2+^ store organelle (Biagini et al., 2003; Rohrbach et al., 2005). Despite the pH-dependency for Ca^2+^ maintenance in the FV, measurement of this ion is challenging, considering the different pH of cellular compartments (Rohrbach et al., 2005). Moreover, the role of FV is associated with hemoglobin degradation (Moura et al., 2009; Tong et al., 2018), chloroquine (CQ) action and CQ-resistance in malaria parasites (Ehlgen et al., 2012; Tong et al., 2018). *P. falciparum* chloroquine resistance transporter (*Pf*CRT), present in the food vacuole membrane (FVM), is apparently very important to balance these processes (Ehlgen et al., 2012; Lee et al., 2018), including its participation in the release of Ca^2+^ from FV (Lee et al., 2018).

The involvement of Ca^2+^ in the functions of FV was initially suggested in *P. chabaudi* by using CQ, where the balance between concentration of intracellular Ca^2+^ and Ca^2+^ in acidic organelles were affected (Gazarini et al., 2007). This could be explained by the parasite’s FV permeability to low-micromolar levels of CQ, leading to Ca^2+^ efflux (Ch’ng et al., 2011). Despite FV potentially playing a role in dynamic intracellular Ca^2+^ storage during asexual intraerythrocytic development (Biagini et al., 2003; Lee et al., 2018), the peculiar metabolic features of this organelle shed light on the possibilities for rational drug design against *Plasmodium* species. For example, a recent report showed that from the 400 Pathogen Box compounds, 10 displayed disruption of FV Ca^2+^ levels comparable to those with CQ, suggesting a compromised FV membrane integrity leading to programmed cell death (PCD) in the parasite (Tong et al., 2018).

A number of reports have discussed new perspectives on acidocalcisomes in parasites. In *T. gondii*, the Ca^2+^/H^+^-ATPase (TgA1) and a vacuolar-type H^+^-pyrophosphatase (TgVP1) are localized in these organelles (Luo et al., 2001; Drozdowicz et al., 2003). Gene disruption revealed that TgA1 is required for polyphosphate storage, intracellular Ca^2+^ homeostasis, microneme secretion, invasion and virulence (Luo et al., 2005). Moreover, Ca^2+^ uptake occurs in these structures by proton pumping activity (Rohloff et al., 2011). In *Trypanosoma brucei*, an IP3R was found in the acidocalcisomes, suggesting that, besides the usual pathway for ER Ca^2+^ release, IP3 can also provide additional regulation for Ca^2+^ mobilization from acidocalcisomes (Huang et al., 2013). Proteomic analysis of this structure in *T. brucei* confirmed the presence of IP3R. The presence of VP1, V-ATPase and vacuolar-Ca^2+^-ATPase (TbPMC1) was also revealed, highlighting evidence of an acidic environment for Ca^2+^ maintenance (Huang et al., 2014). Given the evolutionary evidence of the acidocalcisome (Docampo et al., 2010), it should be not surprising that similar mechanisms may be found in *Plasmodium* species, supporting Ca^2+^ homeostasis in these parasites (**Figure 1**).

### Mitochondrion

Besides the primary role of mitochondria in cellular energy metabolism, they can store Ca^2+^ in both human and murine malaria species (Uyemura et al., 2000). Parasite mitochondria can accumulate part of the Ca^2+^ released in the cytoplasm by pharmacological agents, suggesting a role in maintaining Ca^2+^ homeostasis (Gazarini and Garcia, 2004). Interestingly, melatonin modulates transcript levels of three genes potentially related to mitochondria fusion/fission in *P. falciparum*: FIS1, DYN1 and DYN2 (Scarpelli et al., 2018). Considering that melatonin has already been associated with Ca^2+^ mobilization (Gazarini et al., 2003; Beraldo et al., 2005; Beraldo et al., 2007; Alves et al., 2011; Pecenin et al., 2018), *Plasmodium* mitochondrion fusion/fission could potentially be controlled by Ca^2+^ signaling during the asexual life cycle.

In addition, the expression of the mitochondrial Ca^2+^/H^+^ antiporter gene *pfcha* (or Ca^2+^/H^+^ exchanger, PfCAX) from *P. falciparum* in the *Xenopus laevis* oocystes has been shown to cause Ca^2+^ uptake after the alkalinization of the intracellular environment, suggesting that the out- or inward-directed Ca^2+^ proton movement is pH-dependent (Rotmann et al., 2010). Regarding this feature in Ca^2+^ transport in *P. berghei*, PbCAX expression has been observed in certain sexual stages (gametocytes, zygotes and ookinetes), essential to ookinete forms and parasite transmission to the mosquito *in vivo*, but not essential to the erythrocytic stages of *P. berghei.* A *pbcax* disrupted strain revealed a stage-specific role of this transporter for *Plasmodium* survival (Guttery et al., 2013).

Ca^2+^ disturbance of the *Plasmodium* FV caused by CQ-treatment directly affects the mitochondrial transmembrane potential (Ch’ng et al., 2011; Tong et al., 2018) and triggers a PCD-like phenotype (Tong et al., 2018), providing evidence for Ca^2+^-regulating a functional interplay between *Plasmodium* FV and mitochondria. Additional studies are required to understand the roles of the malaria parasite mitochondrion in Ca^2+^ fluctuation and how CQ affects mitochondrial membrane potential in a Ca^2+^-dependent manner in the FV (**Figure 1**).

## Ca^2+^ Binding Proteins (CaBPs)

CaBPs are conserved among species and present a helix-loop-helix structural motif, known as an EF-hand motif. This motif is generally pair-structured and exposes its calcium-binding domain where two Ca^2+^ ions connect to it. Analysis of the *P. falciparum* genome databank (PlasmoDB) identified 103 potential proteins with EF-hand motifs. Nonetheless, this number is undoubtedly overestimated due to the divergence of the EF-hand motif and some rifins. Without rifins, 43 proteins containing EF-hands were recorded in *P. falciparum* (Lourido and Moreno, 2015). Some reports have proposed that this number is even lower, with about only 30 putative CaBPs (Brochet and Billker, 2016). Three main families of CaBPs are categorized in the Apicomplexa: the calmodulin (CaM) family (including centrins or caltractrins), the calcineurin B-like (CBL) family and the Ca^2+^-dependent protein kinases (CDPK) family (Moreno et al., 2011; Lourido and Moreno, 2015; Brochet and Billker, 2016).

Calmodulin in *P. falciparum* (PfCaM) is localized diffusely in the cytoplasm during mature stages of the intraerythrocytic cycle and at the apical pole end of merozoites within the ductule of rhoptries (Scheibel et al., 1987). Furthermore, a protein kinase B (PfPKB) interacts with PfCaM, which is not a member of the CDPK family, in the schizont/merozoite stages of *P. falciparum*. PfPKB is regulated by PfCaM in a Ca^2+^-dependent manner when the generation of IP3 by PLC mediates Ca^2+^ release. Consequently, PLC is an upstream modulator of PfPKB activity, regulating Ca^2+^ levels inside the parasite and allowing PfCaM-PfPKB interaction (Vaid and Sharma, 2006; Vaid et al., 2008). This protein complex phosphorylates PfGAP45, an anchoring protein of the actin-myosin motor complex from the IMC (inner membrane complex) (Vaid et al., 2008).

**Figure 2:**
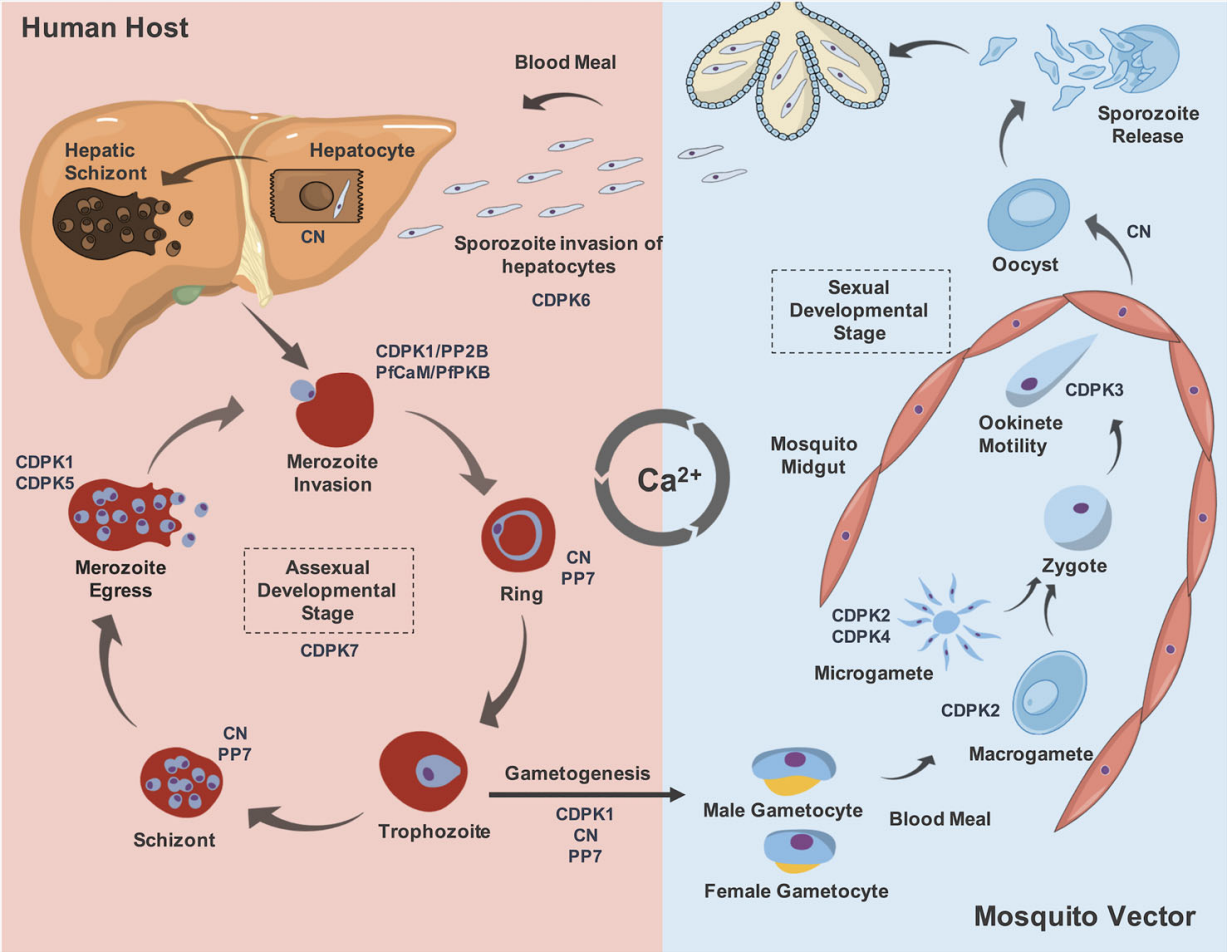
The role of some described Ca^2+^-binding proteins involved in the development stage differentiation and their expression throughout *Plasmodium* life cycle. During the blood meal, the mosquito vector from genus *Anopheles*, inoculate sporozoites released from its salivary glands that will invade hepatocytes. This process is described to be CDPK6-dependent. Moreover, CN allows sporozoite-to-liver stages development inside hepatocytes. Following formation of merozoites in the liver cells, they are released in the bloodstream to continue asexual development stages, invading new RBCs. This process is mediated by CDPK1, CN and PfCaM/PfPKB complex. The role of CDPK7 to maintain the asexual developmental stages is also reported. In addition, the presence of the phosphatases CN and PP7 are implicated in the ring and schizont stages. After schizont maturation, the merozoites are released into the bloodstream to invade new RBCs, which mechanism that requires the action of CDPK1 and CDPK5. Some parasites pass through a morphological transition to form gametocytes, and this process depends on CDPK1, CN and PP7. After a blood meal of the mosquito vector, these forms maturate into male exflagellated and female gametocytes, named as microgametes and macrogametes, respectively. For this transition, CDPK2 and CDPK4 are required. These forms are fused into a zygote, which maturates to a motile ookinete. The ookinete motility is regulated by CDPK3. The ookinete exits from the lumen of mosquito midgut as an oocyst and this transition is mediated by CN. The maturation of the oocyst will release new sporozoites which will migrate into the salivary glands of the mosquito vector. Thus, in an eventual blood meal, these new sporozoites will infect a new host and complete the parasite life cycle in order to propagate malaria disease.

Current understanding is limited regarding a group of four *P. falciparum* centrins (PfCENs 1 to 4: PF3D7_0107000, PF3D7_1446600, PF3D7_1027700 and PF3D7_1105500, respectively), that contain four EF-hand motifs. This group of proteins are involved in parasite cell division at centrosome-like structures, probably in a Ca^2+^-dependent manner (Mahajan et al., 2008). A recent report showed that during mitosis, PbCEN-4 is localized at distinct perinuclear foci, suggesting an association to the putative centrosomal structure, known as the microtubule-organizing center (MTOC) in *P. berghei*. Moreover, *cen-4* gene does not seem to be compensated by increased transcription levels of other centrins and it is dispensable for malaria proliferation (Roques et al., 2019). In contrast, large-scale functional screening of *P. berghei* showed that *cen-1* and *cen-2* genes are essential for parasite survival (Bushell et al., 2017) (**Figure 1**). Other CaBPs and their participation in diverse cellular processes in malaria parasites will be discussed in the next sections (**Figure 2**).

## Ca^2+^-Dependent Protein Kinases (CDPKs)

Protein phosphorylation is one of the most studied post-translational modifications in eukaryotic cellular processes. Regarding the importance of Ca^2+^/CaM in kinase activation, many classes of Ca^2+^/CaM-dependent kinases (CaMKs) are known in mammals. *P. falciparum* protein kinase 2 (PfPK2) is the unique homolog of human functional CaMK that phosphorylates its substrate in a Ca^2+^- and CaM-dependent manner, and it is expressed during invasion (Kato et al., 2008).

However, apicomplexan parasites use a group of Ca^2+^-dependent protein kinases (CDPKs), which are not present in humans. Canonical CDPKs have four EF-hand Ca^2+^-binding domains attached to the C-terminus of a catalytic kinase domain that shows high homology with CaMK. While CaMKs can self-inhibit through a C-terminal helix, CDPKs are regulated by their Ca^2+^-binding domains. In these cases, CDPKs undergo structural and conformational changes, promoting the regulation of other proteins by phosphorylation (Wernimont et al., 2010). *P*. *falciparum* possesses seven CDPKs (PfCDPK1 to PfCDPK7), with correspondent orthologs in plants, but not in animals or fungi (Kadian et al., 2017; Ghartey-Kwansah et al., 2020). It has been proposed that CDPKs could be a novel field for exploration of new antimalarial drugs (Hui et al., 2015).

For instance, transcriptomic data analysis has suggested that PfCDPK1 is primarily expressed in the late schizont stage (Bozdech et al., 2003; Le Roch et al., 2003). In agreement with this, PfCDPK1 is found in the PV and merozoite membrane throughout schizogony and merozoite egress, and performs crucial roles in the invasion process (Azevedo et al., 2013; Bansal et al., 2013). PfCDPK1 is known to phosphorylate both the myosin light chain and an IMC member, PfGAP45, in mature schizonts *in vitro*, when merozoites are formed (Green et al., 2008). As previously reported, PfGAP45 is also phosphorylated by PfPKB (Vaid et al., 2008), but it is proposed that this IMC member is phosphorylated on CDPK1 non-dependent phosphosites (Green et al., 2008). PfCDPK1 knockout mutants showed that this kinase is required for normal growth of *P. falciparum* during asexual proliferation, with critical involvement in gametogenesis, making its transmission to the mosquito unfeasible (Bansal et al., 2018). In contrast, CDPK1 deletion in *P. berghei* showed no difference for the asexual development and host cell invasion, suggesting different functions of the homologs in both species (Jebiwott et al., 2013).

PfCDPK1 mutant parasites on the bulky gatekeeper residue T145M (gatekeeper residue in the wild-type is a Thr, modified to a Met at the position 145 in the mutants) showed prominently reduced activity compared to wild-type parasites. This lower activity seems to be compensated by PKG, influencing the up-regulation of transcription levels of CDPK5 and CDPK6 in the CDPK1 T145M mutant parasites (Bansal et al., 2016), suggesting that a Ca^2+^- based signaling may modulate a very collaborative role in the CDPK family and other kinases in malaria parasites (Green et al., 2008; Brochet et al., 2014). Some reports have highlighted the importance of PfCDPK1 in the phosphorylation of members of IMC, such as GAP45 and IMC1g (Green et al., 2008; Kumar et al., 2017). PfCDPK1 knock-down mutants using the FKBP destabilization domain (DD) showed different patterns of phosphorylation in the protein-partners, revealed by iTRAQ-based phosphoproteomic analysis, including the phosphorylation pattern on S149 of PfPKA, which is a kinase also involved in Ca^2+^-signaling mediated by cAMP (Kumar et al., 2017). Additionally, PfCDPK1 can phosphorylate PfSERA5 (*P. falciparum* serine repeat antigen 5). The PfCDPK1 inhibitor, purfalcamine, blocked SERA5 phosphorylation, leading to the blockage of merozoite egress (Iyer et al., 2018). Despite this evidence on PfCDPK1 as a promising target for therapeutic intervention, a recent chemical genetics approach casts doubt on this suitability for blood stages (Green et al., 2016). Nevertheless, PfCDPK1 continues to represent a good target for a mosquito transmission-blocking strategy, as previously mentioned (Bansal et al., 2018).

In contrast to PfCDPK1, PfCDPK2 function is poorly understood in *Plasmodium*. In all rodent and some other malaria species, the *cdpk2* gene is lacking (Tewari et al., 2010). Initially thought as an essential gene in *P. falciparum*, a recent report has pointed out that in PfCDPK2 knockout mutants obtained by CRISPR-Cas9, it is dispensable in asexual proliferation in *P. falciparum*. Still, CDPK2 seems to play an essential role in male gametocyte exflagellation and possibly in female gametocytes, compromising parasite transmission to mosquitoes (Bansal et al., 2017). Likewise, CDPK4 has been demonstrated to play crucial roles in gametocyte exflagellation (Billker et al., 2004; Ojo et al., 2012). The bumped-kinase inhibitor 1 (BKI-1), which is more than 20-fold more selective for PfCDPK4 over PfCDPK1, inhibited the microgamete exflagellation of *P. falciparum*, but did not block asexual parasite proliferation. A strong correlation between PfCDPK4 activity inhibition and blockage of exflagellation by a series of closely related BKI analogues was observed, supporting that the exflagellation blockage was due to the inhibition of PfCDPK4 rather than other kinases. Furthermore, BKI-1 blocks *P. berghei* transmission to mosquitoes (Ojo et al., 2012).

The apparent role of CDPK4 in the onset of axoneme motility, DNA condensation and cytokinesis during the first 10 min of exflagellation induction has been reported (Fang et al., 2017). An increased interest in the CDPK4 as a new antimalarial target for pyrazolopyrimidine-based inhibitors has also been reported, which could result in new therapeutic strategies for malaria treatment in the near future (Vidadala et al., 2014). Regarding the sexual stages of development, CDPK3 is intimately implicated in regulating the motility of the ookinete in the mosquito vector midgut (Ishino et al., 2006; Siden-Kiamos et al., 2006). *In vitro* migration assays also suggested that this motility is stimulated by Ca^2+^ mobilization from intracellular stores (Ishino et al., 2006).

PfCDPK5 is an important regulator of parasite egress, a highly coordinated event requiring PfSERA5 (Dvorin et al., 2010; Absalon et al., 2018). The egress in CDPK5-deficient merozoites is impaired. PfCDPK5 is localized within micronemes and plays a central role in the micronene protein discharge, correlating a defect in this process to the impaired egress observed in PfCDPK5-deficient parasites. In addition, PKG has been identified as an important protein that cooperates in the egress signaling pathway together with PfCDPK5 (Absalon et al., 2018). This could explain the increased transcriptional expression levels of PKG and PfCDPK5 in the PfCDPK1 mutants as mentioned above, suggesting an integrated cross-talk among kinases in malaria parasites (Bansal et al., 2016), including their role in Ca^2+^ mobilization in gametocyte activation of *P. berghei* and egress of merozoites in *P. falciparum* (Brochet et al., 2014).

Functional studies to understand the roles of CDPK6 and CDKP7 are still lacking. PbCDPK6 has been demonstrated to play a critical role in sporozoite invasion of cells with high expression of heparan sulphate proteoglycans (HSPGs), such as hepatocytes, involving the induction of the circumsporozoite protein (CSP) cleavage upon contact with hepatocytes (Coppi et al., 2007). As previously highlighted, PfCDPK6 could be playing a compensatory role in the asexual blood stages of *P. falciparum* in the absence of a functional PfCDPK1 (Bansal et al., 2016). Still, additional studies need to be performed to address this question adequately. On the other hand, PfCDPK7 is an atypical member of the CDPK family, containing a pleckstrin homology domain adjacent to the kinase domain and two Ca^2+^-binding EF-hands, present at its N-terminus. PfCDPK7 interacts with PIP_2_ through its pleckstrin domain, suggesting that this feature may determine its subcellular localization, possibly at ER exit sites. Moreover, knockout mutants of PfCDPK7 have also shown its importance for the growth of asexual stages of development, presenting abnormal morphology (Kumar et al., 2014). Despite this evidence on the roles of CDPK6 and CDPK7, their downstream signals, which may be implicated in other biological processes, are still largely unknown (**Figure 2**).

## Ca^2+^-Related Phosphatases

Sixty-seven candidate phosphatases were identified in the *P. falciparum* genome by *in silico* analysis (Pandey et al., 2014). At least three serine/threonine protein phosphatases (STPP) are involved in Ca^2+^ signaling: STPP 2B catalytic subunit A (Wilkes and Doerig, 2008; Singh et al., 2014), STPP 7 (PP7) (Kumar et al., 2004; Wilkes and Doerig, 2008; Singh et al., 2014) and a putative STPP 8 (PPP8), which is inferred as containing a Ca^2+^ binding site EF-hand (Yang and Arrizabalaga, 2017; Mitchell et al., 2019).

Calcineurin (CN), also known as STPP 2B or PP3, is a heterodimeric complex containing a catalytic subunit (CNA) and a regulatory subunit (CNB) (Steinbach et al., 2007). CN is conserved from yeast to humans (Yang and Arrizabalaga, 2017) and involved in several cellular processes. It has been extensively studied and reviewed (Crabtree, 2001; Wilkins and Molkentin, 2004; Liu et al., 2015; Park et al., 2019). High Ca^2+^ concentration induces the formation of a Ca^2+^-CaM complex, leading to CN activation, the release of its autoinhibitory domain and exposition of the active site to dephosphorylate its target (Rusnak and Mertz, 2000; Park et al., 2019).

CN in *Plasmodium* spp. is required for host cell attachment and invasion in a receptor-dependent pathway distinct from the AMA1-RON2 (apical membrane antigen-1/rhoptry neck protein 2) system but with some degree of functional overlap (Paul et al., 2015). CN knockdown demonstrated an increase of sensibility to an invasion-inhibitory antibody directed against basigin, an important receptor for RBC invasion, suggesting that CN regulates this process (Duraisingh et al., 2008; Otto et al., 2014; Paul et al., 2015). This might occur regardless of apical organelle proteins involved in invasion (Paul et al., 2015). However, CN has also been described as essential to Ca^2+^-dependent microneme secretion, and its activity is increased after the exposure of merozoites to a low K^+^ environment. The mechanism involving CN and microneme secretion includes regulating apical actin depolymerization (Singh et al., 2014).

Stage-specific conditional degradation of CN in *P. berghei* further demonstrates its role in gametocyte development, fertilization and ookinete-to-oocyst and sporozoite-to-liver stage transitions (Philip and Waters, 2015). CN protein expression and/or activity regulation might provide a regulatory hub during the parasite cell cycle. The protein has been detected at the schizont, ring, sporozoite, merozoite and gametocyte stages, but not in the trophozoite stage (Wilkes and Doerig, 2008; Pandey et al., 2014). Activity inhibition by cyclosporin and FK506 resulted in increased levels of phosphorylated HSP90, phosphoglycerate kinase, actin-1, adenosine deaminase and glyceraldehyde-3-phosphate dehydrogenase. Moreover, actin-1 is potentially a direct substrate of CN in *P. falciparum* (Singh et al., 2014).

Similar to CN, protein phosphatase 7 (PP7) contains EF-hands and IQ (the first two amino acids of the motif: Ile and Gln) calmodulin-binding motifs but, in contrast, is monomeric. The CaM-binding motif was found to inhibit phosphatase activity in *Arabidopsis* PP7 (Dobson et al., 2001; Kutuzov et al., 2001; Yang and Arrizabalaga, 2017). PP7 is not detected at the trophozoite stage but at the schizont, ring, merozoite and gametocyte stages (Dobson et al., 2001; Pandey et al., 2014). These observations indicate that PfPP7 is regulated across all parasite stages and could constitute a potential target to control the parasite cell cycle. PP8 or EFPP is a putative STPP with a long N-terminal domain with EF-hand motifs and is specific to apicomplexans. Mutations were observed in their catalytic domain which put into question their phosphatase activity. Their functions have not yet been investigated (Kutuzov and Andreeva, 2008; Yang and Arrizabalaga, 2017).

Most studies involving the roles of Ca^2+^ signaling and phosphatases are focused on calcineurin. However, other phosphatases without Ca^2+^-binding motifs could be affected by Ca^2+^ signaling through their protein partners possessing these motifs. According to STRING prediction and Gene Ontology analyses, amongst the 67 candidate phosphatases identified in *P. falciparum*, ten potentially interact with proteins involved in Ca^2+^ signaling: putative acid phosphatase (PF3D7_0918000), putative protein phosphatase 2C (PF3D7_0829100), putative 4-nitrophenylphosphatase (PF3D7_0715000), putative protein phosphatase (PF3D7_0802800), putative RNA lariat debranching enzyme (PF3D7_1340600), putative acid phosphatase (PF3D7_1403900), hypothetical protein (PF3D7_1464600), hypothetical protein (PF3D7_1469200), protein phosphatase 2C (PF3D7_0410300) and putative phosphoesterase (PF3D7_1206000) (Pandey et al., 2014). However, the effects of Ca^2+^ on the phosphatase protein-interaction network remain poorly understood in *Plasmodium* species. Therefore, biochemical assays and phosphatase protein partner screenings are a reasonable approach for discovery of new antimalarials (Khalife and Pierrot, 2016).

## Other Effectors Involved in Ca^2+^ Signaling

*P. falciparum* reticulocyte binding-like protein 1 (PfRh1) performs a role in initial sensing of Ca^2+^ followed by signal transduction, causing erythrocyte binding antigen-175 (EBA-175) release from microneme and allowing tight junction formation (Gao et al., 2013). The biochemical pathways regarding Ca^2+^ modulation that led to microneme secretion are largely unknown, highlighting the need for further studies in *Plasmodium* species. As previously mentioned, components of the motor complex involved in merozoite invasion are phosphorylated by PfCDPK1 (Green et al., 2008; Vaid et al., 2008). In *P. berghei* sporozoites, this complex is involved in gliding motility and host cell invasion. Living-cell imaging studies demonstrate that while cytoplasmic elevated Ca^2+^ levels are required for gliding, alone this is insufficient, since artificial increases using an ionophore allowed adhesin translocation to the surface but no gliding motion (Carey et al., 2014). Moreover, the *P. falciparum* inner membrane complex 1l (PfIMC1l) has been proposed as a protein to potentially connect this motor complex to the IMC membrane. It is also involved in gliding and invasion processes in a Ca^2+^-dependent manner. PfIMC1l interacts directly with Ca^2+^ and its interaction with actin is enhanced in the presence of this ion (Kumar et al., 2019). The gliding motility used by ookinete and merozoite invasion is also supported by CDPK4, in a compensatory manner to CDPK1 (and vice versa). Both Ca^2+^-dependent kinases are involved in IMC stability, phosphorylating the glideosome-associated protein 40 (GAP40) and the CDPK4 substrate SOC6 (PBANKA_070770), involved in IMC biogenesis (Fang et al., 2018).

Following microneme secretion, the interaction of PfEBA-175 and the RBCs receptor glycophorin A (glyA) results in a cytoplasmic lowering of Ca^2+^ levels, which, in turn, stimulates the release of rhoptry proteins such as cytoadherence-linked asexual protein gene 3.1 (CLAG3.1/RhopH1) and *P. falciparum* reticulocyte binding-like protein 2b (PfRh2b) (Singh et al., 2010). Rhoptry discharge in RBCs contributes to the tight-junction and PV formation, modifying the host cell environment (Boothroyd and Dubremetz, 2008; Santos and Soldati-Favre, 2011). The repression of *P. berghei* rhoptry neck protein 11 (PbRON11) in sporozoites reduced attachment and motility, leading to the impairment of the infection in the mosquito salivary gland and hepatocyte cells. This protein contains putative EF-hand domains and might act by controlling rhoptry protein secretion in a Ca^2+^-dependent manner (Bantuchai et al., 2019).

Merozoite egress from RBCs is triggered by elevation of cyclic guanosine monophosphate (cGMP) and PKG activation, essential for the protein discharge of secretory organelles, known to support this process (Collins et al., 2013; Alam et al., 2015). Correlation of Ca^2+^ with parasite egress was previously reported (Collins et al., 2013; Glushakova et al., 2013). Events documented in the final hour of the cell cycle include Ca^2+^ release from ER of the schizonts, activation of PfCDPK5 and, in the last 10-20 minutes of the cell cycle, vacuole swelling and red blood cell cytoskeleton destabilization by calpain, a host enzyme activated by Ca^2+^ (Glushakova et al., 2013). More recently, PKG was found to interact with and phosphorylate a multipass membrane protein, termed as important for Ca^2+^ mobilization 1 (ICM1). Conditional knockdown of ICM1 revealed an essential role in Ca^2+^ mobilization to initiate both *Plasmodium* gametogenesis and merozoite egress (Balestra et al., 2021). Additionally, guanylyl-cyclase alpha (GCα)-null mutant parasites were unable to synthesize cGMP for PKG activation in schizonts, leading to a reduction in Ca^2+^ release from internal stores (Nofal et al., 2021).

Conditional gene disruption of the *P. falciparum* phosphodiesterase β (PfPDEβ) leads to a dramatic reduction in schizont cAMP and cGMP hydrolytic activity, resulting in elevated cAMP levels and inappropriate cAMP-induced increased phosphorylation of PKA substrates. In addition, PKA seem to assume a compensatory role with PKG, in order to phosphorylate *P. falciparum* myosin A (*Pf*MyoA), an important component of the so-called glideosome, a parasite complex involved in host cell invasion, in PfPDEβ mutants, bypassing the need for PKG activity by elevated cAMP levels upon Ca^2+^ signaling, possibly by PKA action (Flueck et al., 2019). Together, these findings point towards PfPDEβ regulating cAMP and cGMP production, followed by PKA and PKG activation. Nonetheless, the molecular dynamics of Ca^2+^ signaling associated with these events are still poorly understood.

A family of proteins containing multiple EF-hand motifs, named as the CREC family (calumenin, reticulocalbin 1 and 3, ERC-55, Cab-45), has been remarkably underexplored, considering that proteins from this family are widely found from protozoans to mammals (Honoré and Vorum, 2000). A member of this family is found in the ER of *P. falciparum*, known as PfERC (endoplasmic reticulum-resident calcium-binding protein) (La Greca et al., 1997). This protein is a key regulator of the egress proteolytic cascade of *P. falciparum* merozoites. The use of SERCA inhibitor cyclopiazonic acid (CPA) and an ionophore, ionomycin, did not change the amounts of cytosolic Ca^2+^ in knockdown parasites bearing a glucosamine-inducible ribozyme gene (PfERC-*glmS*) from ER or neutral Ca^2+^ storages, suggesting that the availability of Ca^2+^ from different sources does not change upon knockdown of PfERC. Moreover, PfERC is required for the complete maturation of the aspartic protease plasmepsin X (PMX) in a Ca^2+^-dependent manner, which is required to cleave the subtilisin-like protease (SUB1) (Fierro et al., 2020).

Additional evidence for Ca^2+^ importance for SUB1 discharge and proteolytic cascade events have been reported. Chelation of intracellular Ca^2+^ in *P. falciparum* schizonts blocks the SUB1 discharge from merozoite exonemes into PV, resulting in a decrease of SERA5 proteolytic cleavage and harming PVM rupture and merozoite egress (Agarwal et al., 2013). Mature SUB1 discharge into PV results in the proteolytic cleavage of protein family members involved in merozoite egress and RBC invasion, such as SERA5 and MSP1 (merozoite surface protein 1) (Nasamu et al., 2017; Pino et al., 2017). Additionally to SERA5, SERA6 has been associated to the parasite egress from RBCs upon SUB1 catalytic processing into the PV (Ruecker et al., 2012). In the absence of SERA6, the rupture of RBCM does not occur, suggesting that SERA6 could be associated to an additional proteolytic cascade event related to the β-spectrin cleavage of host cell cytoskeleton (Thomas et al., 2018). Moreover, autocatalytic maturation of SERA6 needs a PV-located protein cofactor, named merozoite surface antigen 180, which is also a SUB1 substrate. This multi-step proteolytic process is required for dismantling the host RBC cytoskeleton facilitating the parasite egress (Tan et al., 2021). Therefore, it remains to be further described how Ca^2+^ may modulate actors in these proteolytic cascade events.

Ca^2+^ signaling has also been shown to be involved in pre-erythrocytic cycle stages. After hepatocyte invasion, elongated sporozoites transform into a spherical form (exo-erythrocytic form, EEF) in a temperature-dependent process (Shortt and Garnham, 1948; Meis et al., 1983; Kaiser et al., 2003). It has been proposed that the Ca^2+^ signal regulates this morphological transition, with intracellular Ca^2+^ increased at the center of a bulbous structure in *P. berghei*, reinforcing that Ca^2+^ plays central roles in diverse life-cycle stages (Doi et al., 2011). Sporozoite salivary gland proteome analyses revealed several components that could be involved in the Ca^2+^ signaling pathway at this stage, such as G-protein-coupled receptors, adenylyl and guanylyl cyclases and a carbonic anhydrase. Host proteins are also involved in EEF transformations and Ca^2+^ signaling. Protein kinase C–mediated NF-κB activation induces expression of CXCR4 (C-X-C chemokine receptor type 4) in hepatocytes and intracellular Ca^2+^ elevation, essential to EEF development (Bando et al., 2019). The interplay between host and parasite proteins, however, remains highly elusive.

## Concluding Remarks

*Plasmodium* species contain distinctive features when compared to other eukaryotes. Such characteristics define its phylum or genus, in which attachment to the host cell, motility, invasion and egress are essential for survival and dissemination. Since Ca^2+^ signaling regulates important and specific *Plasmodium* cellular processes such as microneme secretion, attachment, gliding motility, invasion and egress, actors involved in these pathways, which are regulated by this ion, could be considered potential drug targets. Striking progress to achieve a broader understanding of Ca^2+^ signaling in *Plasmodium* has been made, including the potential involvement of host compounds in Ca^2+^ uptake, such as K^+^, Na^+^, ionic strength and melatonin (Brand et al., 2003; Gazarini et al., 2003; Singh et al., 2010; Pillai et al., 2013; Pecenin et al., 2018).

However, several gaps in understanding remain in these organisms, covering mechanisms involved in increased Ca^2+^ uptaking by iRBCs, together with transport through PVM and the parasite cellular membrane. Moreover, IP3R or alternative functional protein identification in *Plasmodium* would be an important breakthrough to explore Ca^2+^ mobilization and storage, as well as backstage actors which support those processes as promising therapeutic targets. Actually, a plethora of *Plasmodium* proteins with standard and unusual Ca^2+^ binding motifs, which are known or suspected to be involved in Ca^2+^ signaling, could be explored to this end. This also includes proteins without Ca^2+^-binding motifs acting as indirect effectors.

The association of classical techniques employed to study permeability, protein channels and pumps, together with more recent high-throughput approaches is a promise to fulfill these gaps. Mass spectrometry-based proteomics (Garcia et al., 2018; Blomqvist et al., 2020; Garcia et al., 2021), including novel proteomic approaches to understand *in vivo* protein-partners, such as BioID and APEX-2 proximity-labelling techniques (Rhee et al., 2013; Kehrer et al., 2016; Boucher et al., 2018; Birnbaum et al., 2020), metabolomics (Beri et al., 2019) and new Ca^2+^ ratiometric techniques coupled to imaging reporters (Brochet et al., 2014; Carey et al., 2014; Pandey et al., 2016; Absalon et al., 2018; Borges-Pereira et al., 2020) are examples of such technologies required for improved understanding of the role of Ca^2+^ in the backstage of malaria parasite biology and drug screening assay design.

## Author Contributions

All authors contributed to the article and approved the submitted version.

## Conflict of Interest

The authors declare that the research was conducted in the absence of any commercial or financial relationships that could be construed as a potential conflict of interest.

## Publisher’s Note

All claims expressed in this article are solely those of the authors and do not necessarily represent those of their affiliated organizations, or those of the publisher, the editors and the reviewers. Any product that may be evaluated in this article, or claim that may be made by its manufacturer, is not guaranteed or endorsed by the publisher.

## Glossary

**Table:**

IP3R	inositol-1,4,5-triphosphate receptor
IP3	inositol-1,4,5-triphosphate
ER	endoplasmic reticulum
SOCEs	store-operated calcium channels
PMCA	plasma membrane Ca^2+^-ATPase
SERCA	sarco(endo)plasmic reticulum Ca^2+^-ATPase
PLC	phospholipase C
PI(4,5)P_2_	phosphatidylinositol 4,5-biphosphate
RBCs	red blood cells
RBCM	red blood cell membrane
PVM	parasitophorous vacuole membrane
iRBCs	infected red blood cells
uRBCs	uninfected red blood cells
EIPA	ethylisopropyl-amiloride
PTEX	*Plasmodium* translocon of exported proteins
PV	parasitophorous vacuole
cAMP	cyclic-adenosine monophosphate
*Pf*ACβ	*P. falciparum* adenylyl-cyclase β
$\text{HC}{\text{O}}_{3}^{-}$	bicarbonate ion
Epac	exchange protein directly activated by cAMP
Rap1	Ras-related protein 1
*Pf*SR25	G-protein-coupled receptor-like
U73122	inhibitor of PLC
2-APB	2-aminoethyl diphenylborinate, inhibitor of IP3 signaling
PfATP6	a *P. falciparum* SERCA-type Ca^2+^-ATPase
Thg	thapsigargin
FV	food vacuole
PPi	pyrophosphate
PolyP	polyphosphate
VP1	vacuolar-H^+^-pyrophosphatase
V-ATPase	vacuolar-H^+^-ATPase
CQ	chloroquine
PfCRT	*P. falciparum* chloroquine resistance transporter
FVM	food vacuole membrane
TgA1	*T. gondii* Ca^2+^/H^+^-ATPase
TgVP1	*T. gondii* vacuolar-type H^+^-pyrophosphatase
AMDP	amino-methylene-diphosphonate
TbPMC1	*T. brucei* vacuolar-Ca^2+^-ATPase
*Pf*CHA or *Pf*CAX	*P. falciparum* Ca^2+^/H^+^ antiporter or *P. falciparum* Ca^2+^/H^+^ exchanger
CaBPs	Ca^2+^ binding proteins
EF-hand motif	motif for Ca^2+^ binding
CaM	calmodulin
CBL	calcineurin B-like family
CDPKs	Ca^2+^-dependent protein kinases family (1-7)
*Pf*PKB	*P. falciparum* protein kinase B
*Pf*GAP45	*P. falciparum* glideosome-associated protein 45
IMC	inner membrane complex
*Pf*CENs	*P. falciparum* centrins (1-4)
MTOC	microtubule-organizing center
CaMKs	Ca^2+^/CaM-dependent kinases
*Pf*PK2	*P. falciparum* protein kinase 2
PKG	cGMP-dependent protein kinase
PKA	cAMP- dependent protein kinase
IMC1g	inner membrane complex protein 1g
iTRAQ	isobaric tag for relative and absolute quantification
*Pf*SERA5	*P. falciparum* serine repeat antigen 5
HSP90	heat-shock protein
HSPGs	heparan sulphate proteoglycans
CSP	circumsporozoite protein
STPPs	serine/threonine protein phosphatases
CN	calcineurin
CNA	catalytic subunit of the calcineurin
CNB	regulatory subunit of the calcineurin
AMA1-RON2	apical membrane antigen-1/rhoptry neck protein 2
PP7	protein phosphatase 7
IQ	the first two amino acids of the calmodulin binding motif: Ile and Gln
PPP8	serine/threonine protein phosphatase 8
EBA-175	175 kDa erythrocyte binding antigen
*Pf*Rh1	*P. falciparum* reticulocyte binding-like protein 1
*Pf*IMC1l	*P. falciparum* inner membrane complex 1l
GAP40	glideosome-associated protein 40
SOC6	substrate of CDPK4 6
glyA	glycophorin A
CLAG3.1/RhopH1	cytoadherence-linked asexual protein gene 3.1
*Pf*Rh2b	*P. falciparum* reticulocyte binding-like protein 2b
*Pb*RON11	*P. berghei* rhoptry neck protein 11
cGMP	cyclic guanosine monophosphate
ICM-1	important for calcium mobilization-1 protein
cKO	conditional knockout
GCα	guanylyl cyclase alpha
PfPDEβ	*P. falciparum* phosphodiesterase beta
PfMyoA	*P. falciparum* myosin A
CREC family	Calumenin, Reticulocalbin 1 and 3, ERC-55, Cab-45
*Pf*ERC	*P. falciparum* endoplasmic reticulum-resident calcium-binding protein
CPA	cyclopiazonic acid
SUB1	subtilisin-like protease
PMX	plasmepsin X
MSP1	merozoite surface protein 1
EEF	exo-erythrocytic form
NF-κB	nuclear factor κB
CXCR4	C-X-C chemokine receptor type 4
BioID	proximity-dependent biotin identification
APEX-2	ascorbate peroxidase-2
